# Chondrocyte density, proteoglycan content and gene expressions from native cartilage are species specific and not dependent on cartilage thickness: a comparative analysis between rat, rabbit and goat

**DOI:** 10.1186/1746-6148-9-62

**Published:** 2013-04-01

**Authors:** Norazian Kamisan, Sangeetha Vasudevaraj Naveen, Raja Elina Ahmad, Kamarul Tunku

**Affiliations:** 1 Tissue Engineering Group, Department of Orthopaedic Surgery, NOCERAL, Faculty of Medicine, University of Malaya, Kuala Lumpur 50603, Malaysia

**Keywords:** *Rattus norvegicus*, *Oryctolagus cuniculus*, *Capra aegragus hircus*, Native cartilage, Cartilage matrix marker, Glycosaminoglycan

## Abstract

**Background:**

In many pre-clinical studies of cartilage tissue, it has been generally assumed that the major difference of the tissue between the species is the tissue thickness, which is related to the size of the animal itself. At present, there appear to be lack of studies demonstrating the relationship between chondrocyte densities, protein content, gene expressions and cartilage thickness in the various animal models that are commonly used. The present study was conducted to determine whether or not chondrocyte density, proteoglycan/protein content and selective chondrocyte gene expression are merely related to the cartilage thickness (thus animal size), and not the intrinsic nature of the species being investigated. Mature animals (rabbit, rats and goats) were sacrificed for their hind knee cartilages. Image analyses were performed on five consecutive histological sections, sampled from three pre-defined locations at the lateral and medial femoral condyles. Cartilage thickness, chondrocyte density, Glycosaminoglycan (GAGs)/protein content and gene expression levels for collagen II and SOX-9 were compared across the groups. Correlation analysis was done between cartilage thickness and the other variables.

**Results:**

The mean cartilage thickness of rats, rabbits and goats were 166.5 ± 10.9, 356.2 ± 25.0 907.5 ± 114.6 μm, respectively. The mean cartilage cell densities were 3.3 ± 0.4×10^-3 ^for rats, 2.6 ± 0.3×10^-3 ^for rabbits and 1.3 ± 0.2×10^-3 ^cells/μm^2 ^for goats. The mean μg GAG/mg protein content were 23.8 ± 8.6 in rats, 20.5 ± 5.3 in rabbits and 328.7 ± 64.5 in goats; collagen II gene expressions were increased by 0.5 ± 0.1 folds in rats; 0.6 ± 0.1 folds in rabbits, and 0.1 ± 0.1 folds in goats, whilst the fold increase of SOX-9 gene expression was 0.5 ± 0.1 in rats, 0.7 ± 0.1 in rabbits and 0.1 ± 0.0 in goats. Cartilage thickness correlated positively with animals’ weight (R^2^ =0.9856, p = 0.001) and GAG/protein content (R^2^ =0.6163, p = <0.001). Whereas, it correlates negatively with cell density (R^2^ = 0.7981, p < 0.001) and cartilage gene expression levels (R^2^ = 0.6395, p < 0.001).

**Conclusion:**

There are differences in the composition of the articular cartilage in diverse species, which are not directly dependent on the cartilage thickness of these animals but rather the unique characteristics of that species. Therefore, the species-specific nature of the cartilage tissue should be considered during any data interpretation.

## Background

The use of animal experimental models provides a framework of understanding of the pathogenesis, progression and development of a disease, which invariably helps researchers to investigate various potential treatment modalities [1-6]. Of the many translational research involving animal models, studies on the pathogenesis of cartilage disease is of great importance and has been extensively carried out in view of its great implication in further management of osteoarthritis, a disease that is associated with a huge medical and economic burden to the nation [3,7-9]. Like any other translational research, the use of an animal model for studying osteoarthritis must satisfy several requirements, which include the ability to reproduce the disease model, easy to handle, provide sufficient amount of tissue for evaluation and is cost-effective [10]. Weight, age, animal species, surgical technique used, cage activity and exercise will significantly influence the outcome of any animal studies [2]. Smaller animals such as rats and guinea pigs are easier to handle than larger ones. For this practical reason, they are better suited as experimental models in many studies. On the other hand, due to their relatively small size, these animals may provide insufficient amount of samples, particularly for histomorphometry and biochemical analyses. This in turn may result in limited accuracy of the data, creating the need of using a larger pool of animals, which would involve higher cost and more animal sacrifice. Hence, some authors would prefer using larger animals such as rabbits, canines, pigs, goats and various bovine species. Furthermore, the use of larger animals would permit better visualization of the anatomical structures being investigated. More importantly, because of its larger size, it is assumed that these animals would have better clinical relevance to human conditions [3,5].

In many animal studies, it has been generally assumed that that the major difference in articular cartilage between the various species is the tissue thickness, which is related to the size of the respective animal itself. The general assumption is that the larger the animal, the thicker the cartilage, and therefore, there would be higher composition of the articular cartilage including the amount of chondrocytes and its associate extracellular matrix. However, in a previous study, an inverse relationship was found between chondrocyte density and cartilage thickness in a normal sheep articular cartilage [11]; similar observation was also found in normal human femoral head [12,13]. This appear to be a rather paradox phenomenon from the previous general assumption. At present, there are only very few publications involving normal cartilage, and the available data comparing the different species are somewhat limited. There is a recent report on cartilage thickness and chondrocyte density in various species, which formed an integral dataset for establishing a consensus of histopathological scoring systems for most commonly, used osteoarthritis animal models [14]. However, data on other important composition of normal articular cartilage, that is the extracellular matrix such as glycosaminoglycans per protein content and cartilage gene expression levels between different species are not widely available. Therefore, there is need for establishing a more comprehensive dataset that includes major compositions of a normal articular cartilage (i.e. chondrocytes and its associated extracelullar matrix) between various osteoarthritis animal models as reference value for further studies. In addition and more importantly, the question of whether or not chondrocyte density, proteoglycan/protein content and selective chondrocyte gene expression are related to only cartilage thickness and not the nature of species being investigated remains unanswered, as this was not specifically addressed by any other previous studies. This has an implication on data interpretation, such that if the composition of articular cartilage is not dependent on cartilage thickness, the assumptions that larger animals would have better clinical relevance to human conditions may no longer be valid, and that the nature of specific species has to be taken into account when interpreting the data, since the cartilage of one species behaves differently from another. It is reasonable to assume that the increase in cell numbers would correlate proportionately to the increase cartilage thickness. It is worth noting that in previous studies, it appears that the differences in cartilage thickness between animals has no direct correlation with their cell densities and that cell density is more likely to be species specific [15]. However, considering that cartilage of any animal would have similar functions regardless of its size, there should not be apparent discrepancies in the GAGs/protein/cell and selected cartilage genes. This however has not been demonstrated in previous studies. The present study was therefore conducted to determine the cartilage thickness, chondrocyte densities, Glycosaminoglycan (GAGs)/protein content and the selective cartilage gene expression (collagen type II and SOX-9) in the 3 commonly used osteoarthritis animal models: Sprague-Dawley rats (*Rattus norvegiens*), New Zealand white rabbits (*Oryctolagus cuniculus*) and Boer goats (*Caprus hircus*).

In addition, in order to determine whether the differences in articular cartilage across all species are merely related to the cartilage thickness (thus the size of the animal) or the intrinsic nature of the individual species, the relationships between cartilage thickness and the various compositions of articular cartilage in all species were also studied.

## Methods

### Animals

All procedures were conducted with the approval of and in accordance to the guidelines for animal handling and welfare set by the animal ethics committee of University of Malaya (OS/05/08/2009/WJH(R)). Thirty nine animals were used in the study i.e. adult rats (N = 18) (*Rattus norvegicus (200–220 gms)),* rabbits (N = 18) (*Oryctolagus cuniculus (2000-2400gms)*) and goats (N = 3) *(Capra aegragus hircus (37–39.5 kg)*) were obtained from the animal experimental centre of the institution. The weight and age of animals were standardized to closely approximate each other to minimise error. Rats and rabbits were further grouped into three small subgroups consisting of six animals (n = 6) for each of the 3 different analyses. In view of a more abundant cartilage tissue, each sample from the 3 goats was divided into 3 sections for the 3 different analyses.

### Histological evaluation

Animals were euthanized and the knee joints were dissected to obtain the distal femur. Samples were decalcified in 10% formic acid for 11 to 14 days for rats, 21 to 30 days for rabbits and goats. For rats and rabbits, the distal femur was cut into halves at the intercondylar region and labeled as medial or lateral condyle. Samples were dehydrated in ethanol series immersion and embedded in paraffin. An average of ten sections (6 μm thick per section) was cut at regular interval to get a strip of paraffin film with the tissue at the centre of the film. For each block, a minimum of ten slides with at least three samples of tissue per slide was obtained to minimise sampling errors. The slides were divided equally for haematoxylin-eosin (cellular architecture) and Safranin-O-fast green staining (proteoglycan contents of matrix).

### Morphometric analysis

Image analyses were performed (Lumen*era* INFINITY ANALYZE®) on five consecutive histological sections (10 μm thickness and 100 μm^2^ area of interest) obtained from the articular cartilage, sampled at three pre-defined locations (anterior, posterior, weight bearing) of the lateral and medial femoral condyles, respectively. Cartilage thickness and chondrocyte density were manually measured using the software toolkits.

### Cartilage thickness and chondrocyte density

Point-to-point measurement of the articular surface to the subchondral bone was performed using a calliper to determine the cartilage thickness. The measurement was expressed in micrometres. The surface area in each anatomical region was established using a grid superimposed onto the captured image to determine the chondrocyte density. The micrometre was calibrated to 100 μm in length per box. The surface area was measured within the 500 μm (five grid boxes) by measuring the perimeter of the region of interest using the image analysis software. The chondrocytes numbers, which were manually calculated within the area, were divided by the surface area to indicate the chondrocyte density in the cartilage tissue.

### Biochemical analysis

Protein and glycosaminoglycan (GAGs) were determined using Bio-Rad DC protein assay kit (Bio-Rad Laboratories; USA) and Blyscan sulfated Glycosaminoglycan assay kit (Biocolor Ltd., UK) according to the manufacturer’s protocols. Spectrophotometer absorbance measurements were performed at 750 nm and 656 nm for protein and GAGs assays respectively. GAGs content was normalized according to the protein contents (μg GAGs/mg protein).

### Total RNA extraction, cDNA synthesis and real-time PCR

Total RNA was isolated using a homogenizer and then processed according to the cartilage RNA isolation kit (Biochain) protocol. RNA samples were finally re-dissolved in 30 μl water and stored at −20°C. 1 μg of RNA was used to generate cDNA with the Superscript III first strand synthesis kit (Invitrogen, Malaysia) in accordance to the manufacturers’ instructions. Real-time PCR analysis (CFX96 Real-time system, BIO-RAD) was performed to assess the mRNA levels using iQ-SYBR green supermix (BIO-RAD). The data was normalized using Beta Actin (rat)/ GAPDH glyceraldehyde-3-phosphate dehydrogenase (rabbit and goat).

For each target gene (Collagen II and SOX-9), the measured fluorescence following each amplification cycle demonstrated typical profiles: the emitted signal remained at baseline levels during early cycles, followed by an exponential increase in levels. The linear correlation between the Ct values (threshold values) and the initial cDNA amounts, diluted to a certain fold, confirms the accuracy of the method within a wide working range. The amplification efficiency for each target sequence (Et) was normalized to the housekeeping genes (GAPDH (rabbit and goat)/Beta actin (rat)). The target genes were chosen on the basis of their role as chondrocyte marker. COL2A1 gene is an early cartilage matrix marker; and SOX-9, a transcription factor of the SRY family is highly expressed in proliferating chondrocytes at the prehypertrophic zone.

The resulting values, although normalized to housekeeping genes, are still dependent on the efficiency of fluorescence emission. Therefore, the reported figures cannot be considered as absolute expression levels of the genes of interest, and can only be used as relative quantification amounts. GAPDH was chosen as the reference housekeeping gene based on its use in the majority of previous studies on chondrocyte gene expression.

### Statistical analysis

Statistical analysis was carried out only on the rat and rabbit groups, which have comparable number of biological samples. The difference in each parameter between the 2 groups was determined using non-parametric analysis i.e. Kruskal-Wallis test. If values were significant, Mann Whitney U (for dependent variables) and Wilcoxon signed rank (for non-dependent variables) tests were used to evaluate the level of significance between the groups. P values of less than 0.05 were considered significant. To determine the relationships between the histomorphometric parameters, weight, GAGs and gene expression, Pearson correlation test were employed followed by regression analysis on any significant relationships. Our preliminary analyses using both linear and non-linear model showed that the relationships between the various parameters were better predicted by the non-linear regression line (relatively higher R^2^ values). All data were subsequently fitted using a non-linear regression model, which is also deemed to be more appropriate and commonly employed to fit most biological data [16,17]. All analyses were conducted using the SPSS statistical software version 17.0.

## Results

### Histomorphometric results: cartilage thickness

**Figure 1:**
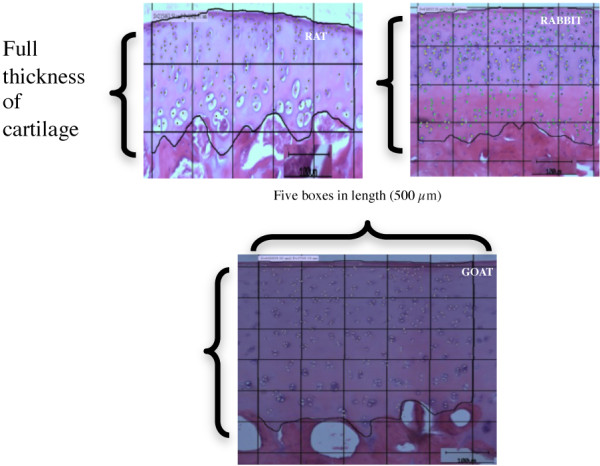
**Cartilage Thickness.** Measurement of cartilage thickness of (**A**) rat, (**B**) rabbit and (**C**) goat. Mean cartilage thickness was derived from a minimum five readings in each slide. Representative slides were obtained from the posterolateral condyle of left distal femur. Surface area is calculated in 500 μm (five boxes) in length and full thickness in depth. (Safranin O staining; magnification rat and rabbit 10×, goat 4× objective lens).

**Figure 2:**
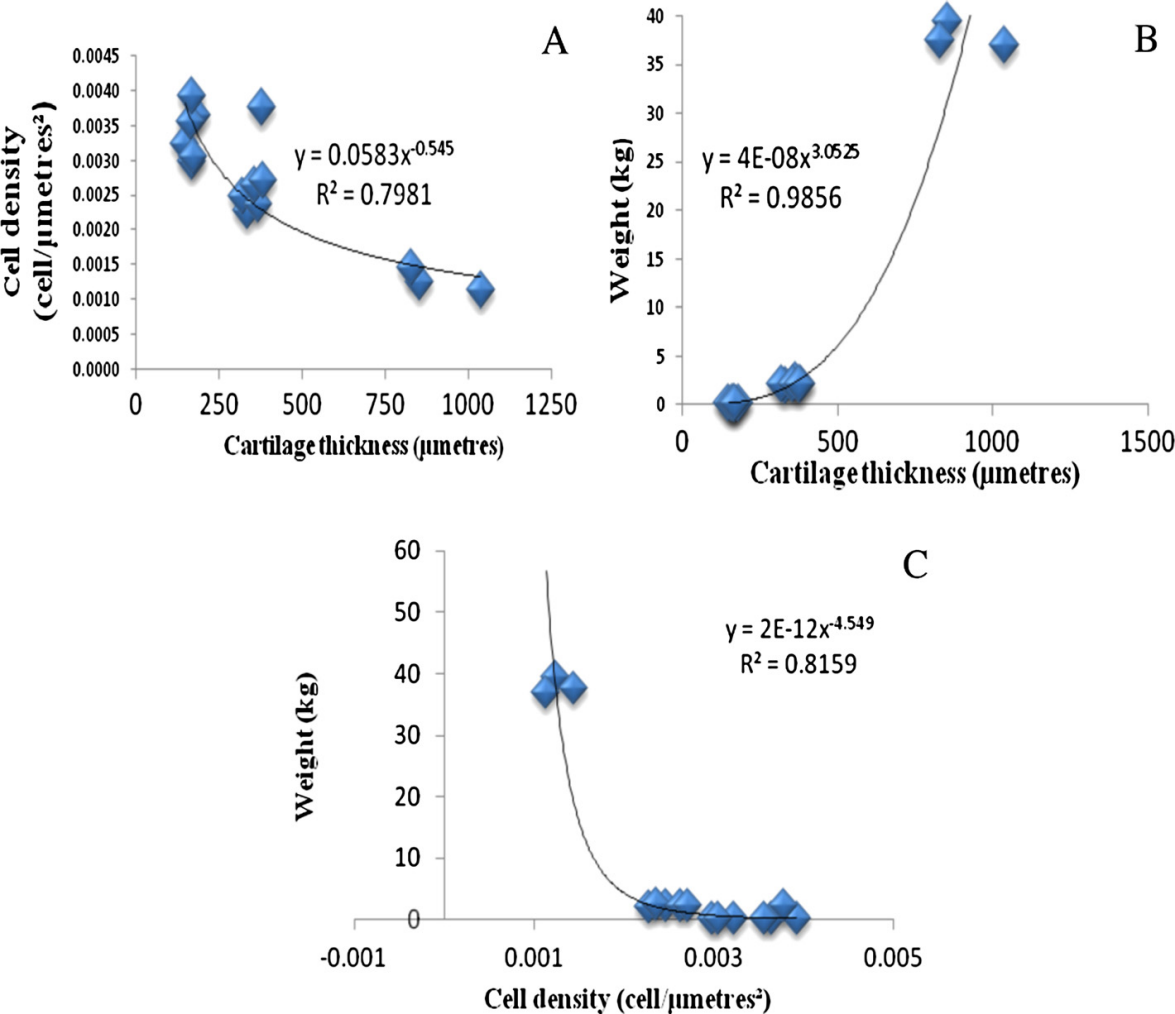
**Correlation for cartilage thickness, cell density and animal weight.** The figure demonstrates: (**A**) An inverse relationship between the cartilage thickness and the cell density. (**B**) Positive correlation of the cartilage thickness and the animal weight. (**C**) An inverse relationship between chondrocytes density and animal weight.

The surface of the articular cartilage was smooth in all animals. The zonal distribution of Safranin O staining in the cartilage was similar in all anatomical locations. In all animals, the staining was weak in the superficial zone and increased towards the deep cartilage zone, in keeping with a normal distribution of proteoglycan in a normal cartilage (Figure 1). The measurement of cartilage thickness is illustrated in Figure 2. Goat has the highest average cartilage thickness (907.5 ± 114.6 μm), followed by rabbit (356.2 ± 25.0 μm) and rat (166.5 ± 10.9 μm)

### Chondrocyte density

The superficial zone of the articular cartilage of rat and rabbit contained densely arranged flattened disc-like chondrocytes; the cells appeared to be less dense in the goat articular cartilage (Figure 1). All animals demonstrated similar pattern of chondrocytes morphology in the middle and deep zones; the chondrocytes appeared more rounded in the middle zone and arranged in columns in the deep radial zones, particularly in the posterior and weight bearing regions. The cartilage cell densities of the animals were 3.3 ± 0.4×10^-3 ^cells/μm^2 ^for rat, 2.6 ± 0.3×10^-3^ cells/μm^2 ^for rabbit and 1.3 ± 0.2×10^-3^ cells/μm^2 ^for goat.

### Correlation between chondrocytes density, cartilage thickness and weight

There was a statistically significant positive correlation between cartilage thickness and weight of all animals (Figure 2(B)) (R^2^ = 0.9856, p < 0.001). An inverse relationship was observed between chondrocytes density and cartilage thickness, as shown in Figure 2(A) (R^2^ = 0.7981, p < 0.001). In addition, a significant negative correlation was observed between chondrocytes density and the weight of all animals (R^2^ = 0.8159, p < 0.001) (Figure 2(C)).

### Biochemical analysis

**Figure 3:**
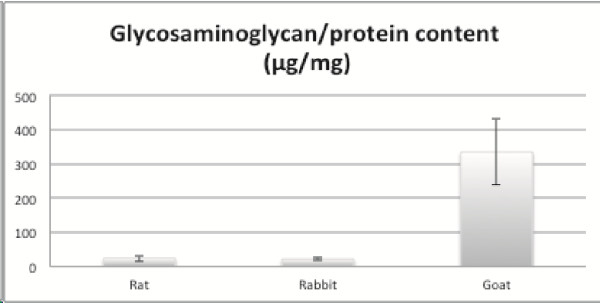
**Comparison in the Glycosaminoglycan/protein content.** Goat cartilage contained the highest amount of GAGs/protein (μg/mg) followed by rat and rabbit.

GAGs/protein (μg/mg) content was higher in goat (328.7 ± 64.5), followed by rat (23.8 ± 8.6) and rabbit (20.5 ± 5.3) (Figure 3). No significant difference in GAGs/protein content was observed between the rat and rabbit group.

### Correlation between GAGs/protein content and animal weight/cartilage thickness/cell density

**Figure 4:**
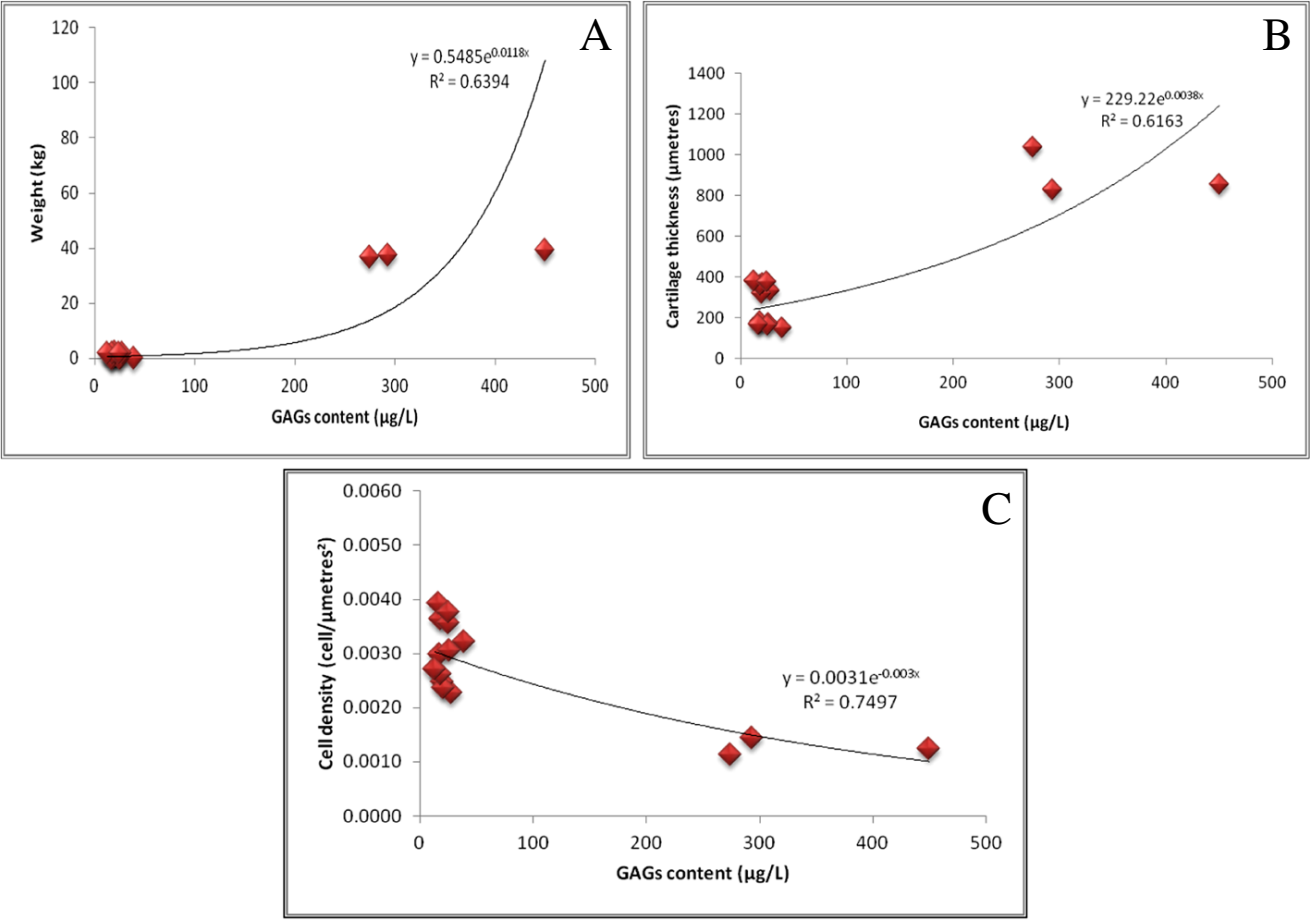
**Correlation for GAGs/protein content and cartilage thickness/cell density/animal weight.** Figures demonstrate: (**A**) GAGs/protein and the weight of the animals. (**B**) GAGs/protein and cartilage thickness. (**C**) GAGs/protein and cell density.

There were significant correlations between the GAGs content and weight (, R^2^ = 0. 6394, p < 0.001) (Figure 4A), cartilage thickness (R^2^ = 0.6163, p < 0.001) (Figure 4B), cell density (R^2^ = 0.7497, p = 0.001) (Figure 4C). The trend line in Figure 4A indicates that large animals are associated with higher amount of GAGs as compared to small animals. The scatter plot in Figure 4B demonstrates that thicker articular cartilage had larger amounts of GAGs per protein content as compared to thinner cartilage. Rabbit with mean cartilage thickness of 450 μmetres had lower amount of GAGs as compared to rats. It is interesting to note that the higher amounts of GAGs were found in cartilage with lesser cell density (Figure 4C). These correlations however, do not appear to be linear.

### Gene expression analysis

**Figure 5:**
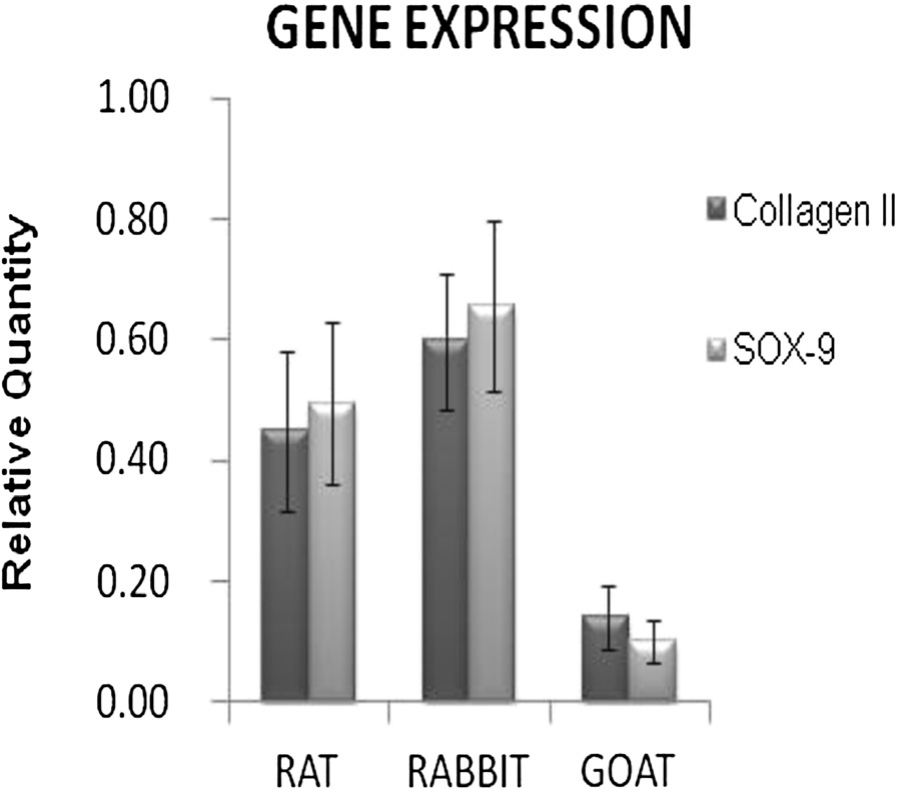
**Gene expression analyses.** Gene expression analysis of both collagen II and SOX9 in rat, rabbit and goat demonstrate significant variation between species.

Comparative analysis of cartilage genes expressions from all animals (Figure 5) showed that the samples from goat had a very low concentration of RNA, and therefore, the expression of collagen II and SOX-9 have decreased considerably when compared with rat and rabbit.

### Correlation between collagen II and SOX9 gene expression and animal weight, cartilage thickness, cell density and GAGs/protein content

**Figure 6:**
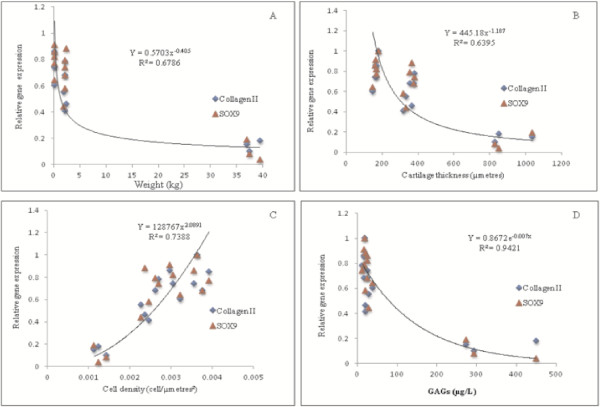
**Correlation for gene expression pattern.** Correlation of gene expression patterns for Collagen II and SOX-9: (**A**) An inverse relationship was observed between the genes expressed and animal weight. (**B**) Thicker cartilage had low levels of gene expression as compared to thin articular cartilage as depicted by a downward non-linear line in this graph. (**C**) The upward line in the graph demonstrates that cartilage with less cell count per surface area has low levels of gene expression, possibly due to the low concentration of RNA in the chondrocytes of the articular cartilage. (**D**) The inverse line in the graph suggests that cartilage with low level of gene expression had greater amounts of GAGs/protein.

There was a statistically significant negative correlation between collagen II and SOX9 gene expression with animal weight (R^2^ = 0.6786, p < 0.001) (Figure 6A). Consistent with that, a significant negative correlation was also found between collagen II and SOX9 gene expression and cartilage thickness (R^2^ = 0.6395, p < 0.05) (Figure 6B). On the other hand, a significant positive correlation was observed between the gene expression level and cell density of all animal species (R^2^ = 0.7388, p < 0.001) (Figure 6C), which indicates that higher level of gene expression was found in the cartilage with increased cell density.

## Discussion

The present study suggests that cell density, GAGs/protein content and chondrocyte candidate gene markers is not directly dependent on the thickness of the animal cartilage but rather the species from which the cartilage is derived. The study showed that compared to smaller animals, the larger ones had thicker cartilage but lower chondrocyte densities and lower cartilage gene expression (Collagen type 2 & SOX-9). This has strong implications to many studies using animals as a representative clinical translational model. It was our initial assumption that although the increase in cartilage thickness has to correlate with the increase in animal size e.g. goat cartilage is thicker than rats; it is unlikely that cell density will differ when we consider that the size of chondrocyte of any mammal should remain similar, especially in quadrupeds [15,18]. The present study also showed that weight and size of the animals play a significant role in maintaining cartilage thickness, a fact that is supported by several published literatures [19-21]. The extent of the mechanical stimuli related to the weight of the animal may contribute to the proportional increase in the thickness of articular cartilage. Furthermore, it may also be argued that genetic factor may contribute to the differences in cartilage thickness between various animals by altering the intrinsic properties of the articular cartilage itself. This however has not been substantiated by any previous studies. It is however worth noting that cartilage thickness changes with age, peaking in maturity but undergo continual degradation thereafter, albeit minimally [12,13,22].

The number of chondrocytes in this study is apparently higher as the cartilage becomes thinner, an observation that is also supported by other studies [11,12]. Furthermore, in this study, the cell contents decreased towards the deeper parts of the cartilage (Figure not shown). In articular cartilage, it has been previously indicated that changes in cell density from the surface to the inner depth are mediated by factors that act on the articular surface such as nutritional supply and mechanical stress [12]. In the present study, cell densities followed an increasing pattern from goat, rabbit, and rat with a ratio of 1:2:3 cells/μm^2^. A possible explanation for this may be that chondrocytes of the smaller animals’ exhibits an increase in cartilage metabolism, which in turn increases the cell turnover to maintain cartilage integrity. This has been suggested by previous studies [22,23]. Another factor which may account for the variation in cell density is aging [13]. It has been reported that one of the possible mechanisms that is responsible for the loss of the cells in aging could be apoptosis, which may be evident as decreased cell density [23]. However, the loss of cells that takes place in natural aging is not expected to be too significant to the extent of compromising the integrity of the cartilage tissue on the whole [24]. In the present study, the difference in chondrocytes density across species may not have been well explained by aging since the selection of all animals were standardised to the maturing age of the animal [22,25]. A major strength of the study is the provision of a more comprehensive data on the composition of the normal articular cartilage, which not only include chondrocytes density but also the extracellular matrix protein (glycosaminoglycan or GAGs) and cartilage candidate gene expressions (Collagen type II and Sox-9). The present study demonstrates that smaller animals such as rats exhibited lower amounts of GAGs compared to larger animal such as goat, consistent with the findings of others [26,27]. These findings appear to support a logical assumption that the increase in animal size provides a high GAGs/protein content in the articular cartilage.

It is hypothesized that an increase in GAGs is related to some extent to the increase in cartilage thickness. A possible explanation for this may be that an increase in mechanical loading of cartilage leads to an increase in glycosaminoglycan content, which may improve cartilage stiffness and resiliency [28-30]. The adaption of cartilage to loading may be one several mechanisms that reduces the risk of developing cartilage degeneration at sites which are subjected to high amounts of compressive loading. This process results in both an increase in cell protein expression and cell proliferation [31-33]. However, the increase in GAGs content in this case may not necessarily be attributed to a direct increase in its production in proportion to the increase in cell numbers. In fact, in the present study, an inverse relationship exists between GAGs content and cell density, suggesting that individual chondrocyte may have been producing more GAGs per cell in response to heighten mechanical stresses, as opposed to a higher amount of GAGs being produced by larger cell numbers. In the second scenario, individual chondrocyte would seem to be producing similar amount of GAGs per cell. This also indicates that normal mechanical stress may not stimulate cell replication as demonstrated in previous studies [34].

This study also examines the chondrocyte specific genes i.e. collagen II and SOX-9 that were naturally being expressed within the different species. It was evident that cartilage of smaller animals (rats) had the highest level of collagen II and SOX-9 gene expression compared with the goat cartilage. This finding was noted to be reflected by the greater chondrocytes density in the small animals. Higher chondrocytes count per surface area indicates a higher level of mRNA concentration within the matrix, consistent with an increase in the production of collagen in these animals. Although the expressed collagen II gene was lesser in goat cartilage, the highest amount of GAGs was observed in this animal. A further question arises concerning the inverse relationship between collagen II and GAGs content. Caution should be exercised when interpreting this result, as the increase in gene expression may not necessarily mean an increase in protein that is being translated. Unfortunately, it was not possible to determine if this was the case in the present study. Moreover, there has not been any study that has explored this relationship previously. Thus, the contrasting observation between GAGs/protein and cartilage gene expression levels observed in this study remains to be explained and further explored by future studies.

Many studies that describe the cartilage repair outcomes *in vivo* in animal model of osteoarthritis [35-37] are based on the assumption that chondrocyte function remains the same in all of the animals used in the experiments. It is assumed that smaller animals such as rabbits tend to heal faster. However, no studies have demonstrated that smaller the animal, there would be an increase in the gene expression of cartilage specific proteins e.g. proteoglycan that would promote chondrogenesis and expedite the healing process. The present study addresses these issues and now provides supporting evidence that this is not the case, but rather, chondrocyte function is unique to the species from which the tissue is obtained. This finding also indicates that data published in *in vitro* experiments using cells of various animal species have to be interpreted with caution since chondrocyte metabolism from various species are inherently different. Our study demonstrates that chondrocyte function in rabbit (in terms of the protein and gene expressed) is actually higher than the other two species, supporting the notion that irrespective of the size of the animals (and thus the thickness of their cartilage), the cells themselves have an intrinsic characteristic, metabolism and function that is unique to the individual species.

## Conclusions

This study demonstrated the differences in the composition of the articular cartilage in three commonly used osteoarthritis animal models of varying sizes. These differences are not directly dependent on the animal size, and thus their cartilage thickness, but rather, the cell behaviour is likely to be related to its unique inherited trait, being species specific. Therefore, cautions should be exercised when interpreting data from animal models in translational research that aims at human application.

## Competing interest

The authors declare that they have no competing interests. The authors have no financial or personal relationships with other people or organizations that could inappropriately influence or bias the contents of this paper.

## Authors’ contributions

TK, KN and NVS conceived and designed the study; KN and NVS collected and analyzed the data; REA assisted in data collection methods and TK, NVS and REA helped draft the manuscript; all authors read, contributed to and approved the final manuscript.
