# Correction: Chondrocyte density, proteoglycan content and gene expressions from native cartilage are species specific and not dependent on cartilage thickness: a comparative analysis between rat, rabbit and goat

**DOI:** 10.1186/1746-6148-9-136

**Published:** 2013-07-10

**Authors:** Norazian Kamisan, Sangeetha Vasudevaraj Naveen, Raja Elina Ahmad, Tunku Kamarul

**Affiliations:** 1 Tissue Engineering Group, Department of Orthopaedic Surgery, NOCERAL, Faculty of Medicine, University of Malaya, Kuala Lumpur, 50603, Malaysia

## Correction

After the article was published [1], we became aware that the name of our corresponding author was not correct. It should have been Tunku Kamarul [2,3] AND NOT Kamarul Tunku.

We hope that you are able to make the necessary amendments to this error and apologize for any inconvenienced caused as the result of this misprint.
